# Tris(aminomethyl)phosphines and Their Copper(I) (Pseudo)halide Complexes with Aromatic Diimines—A Critical Retrospection

**DOI:** 10.3390/ph16050766

**Published:** 2023-05-19

**Authors:** Radosław Starosta

**Affiliations:** 1 Faculty of Chemistry, University of Wroclaw, F. Joliot-Curie 14, 50-383 Wroclaw, Poland; radoslaw.starosta@uwr.edu.pl; 2 Centro de Química Estrutural, Institute of Molecular Sciences, Faculdade de Ciências da Universidade de Lisboa, Campo Grande, 1749-016 Lisboa, Portugal

**Keywords:** copper(I), tris(aminomethyl)phosphines, metal complexes, antibacterial activity, antifungal activity, anticancer activity, plasmid DNA, serum albumin

## Abstract

Metal complexes feature a wide range of available geometries, diversified lability, controllable hydrolytic stability, and easily available rich redox activity. These characteristics, combined with the specific properties of coordinated organic molecules, result in many different mechanisms of biological action, making each of the myriads of the classes of metal coordination compounds unique. This focused review presents combined and systematized results of the studies of a group of copper(I) (pseudo)halide complexes with aromatic diimines and tris(aminomethyl)phosphines of a general formula [CuX(NN)PR_3_], where X = I^−^ or NCS^−^, NN = 2,2′-bipyridyl, 1,10-phenanthroline, 2,9-dimethyl-1,10-phenanthroline or 2,2′-biquinoline, and PR_3_ = air-stable tris(aminomethyl)phosphines. The structural and electronic properties of the phosphine ligands and luminescent complexes are discussed. The complexes with 2,9-dimethyl-1,10-phenanthroline, apart from being air- and water-stable, exhibit a very high in vitro antimicrobial activity against the *Staphylococcus aureus* and *Candida albicans*. Moreover, some of these complexes also show a strong in vitro antitumor activity against human ovarian carcinoma cell lines: MDAH 2774 and SCOV 3, CT26 (mouse colon carcinoma), and A549 (human lung adenocarcinoma) cell lines. The tested complexes are moderately able to induce DNA lesions through free radical processes, however the trends do not reflect observed differences in biological activity.

## 1. Introduction

The success of cisplatin and its analogs in clinical cancer treatment [1] paved the way to the exploration of metals, metal ions, and coordination compounds in almost all kinds of biomedical applications. What distinguishes metal-based agents from organic drugs is not only a wider range of available geometries, diversified lability, and controllable hydrolytic stability, but also easily available rich redox activity. These features, combined with specific properties of coordinated organic molecules, result in a wide range of mechanisms of biological action, making each of the myriads of the classes and even groups of metal complexes unique, as shown in recent general reviews [2,3,4,5] and reviews focused on copper complexes [6,7]. This focused review is the first attempt to comprehensively systematize the fragmented data describing the physicochemical properties and biological activity of copper(I) complexes with tris(aminomethyl)phosphines and aromatic diimines from several articles [8,9,10,11,12,13,14,15,16,17].

**Figure 1:**
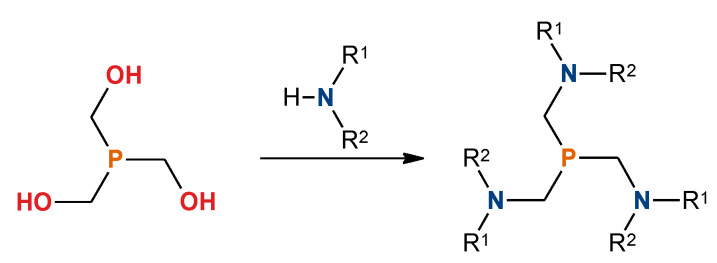
Schematic representation of the synthesis of tris(aminomethyl)phosphines.

The synthesis of tris(aminomethyl)phosphines from tris(hydroxymethyl)phosphine and dialkylamines (Figure 1) was first described by Coates and Hoye in 1960 [18]. Since then, the chemistry of this class of compounds has been developed systematically, resulting in a large number of tris(aminomethyl)phosphines synthesized and characterized [19,20].

Indeed, a practically infinite range of possible aminomethylphosphines and their structural diversity, which can lead to multiple applications in chemistry, biochemistry, and biomedical sciences, encouraged us to study this class of compounds. When we started to work on this project, there were almost no studies of the coordination chemistry of tris(aminomethyl)phosphines in the literature, with only a few metal complexes with these ligands published until 2010: polynuclear copper(II) complexes with P(CH_2_NHC_6_H_3_-3,5-(CF_3_)_2_)_3_ [21], magnesium complexes with P(CH_2_NHC_6_H_3_-3,5-(CF_3_)_2_)_3_ or P(CH_2_NHPh)_3_ [22], in which phosphines act as multidentate bridging ligands coordinating via nitrogen atoms and a series of rhodium complexes with the P(CH_2_NHAr)_3_ (where Ar = Ph, C_6_H_3_-3,5-(CH_3_)_2_, C_6_H_3_-3,5-(CF_3_)_2_) additionally internally coordinating titanium atoms via nitrogen atoms [23].

The goal of the undertaken studies was to determinate the relationships between the structures and widely understood activity in the copper(I) complexes with tris(aminomethyl)phosphines and aromatic diimines. We chose the copper(I) cation for several reasons. Primarily, copper, being an essential trace element, should be less toxic than non-endogenous metals. Therefore, designing therapeutic agents based on this metal seems applicable. Although copper has a long history of application in medicine, copper(I) complexes have not been examined extensively until recently, and a few Cu(I) complexes with phosphines showed promising biological activity [24,25,26,27,28,29,30]. Notably, while the Cu(II) ion is an intermediate (soft–hard) acid, the Cu(I) ion is a soft acid with a preference for soft bases, such as sulfur- and phosphorus-based ligands. Additionally, it has been proven that the use of water-soluble ligands is plausible, since their hydrophilic and labile complexes are more active than the complexes with chelating, lipophilic diphosphines [31,32]. Furthermore, studies of Cu(I) mixed-ligand complexes with diimines and aromatic phosphines or diphosphines ([Cu(NN)(P)_2_]^+^ or [Cu(NN)(P_2_)]^+^) [33,34,35,36,37,38,39,40,41,42,43,44] are very extensive, because of their diversified photophysical properties. Nevertheless, studies on neutral heteroleptic complexes of [CuX(NN)P] type, where X = halogen or pseudohalogen, P = phosphine, and NN = diimine, were scarce. Rather limited papers focused mainly on luminescent properties of [Cu_2_X_2_(PPh_3_)_2_] or [CuX(PPh_3_)_3_] (X = I, Br, Cl) complexes [45], [Cu_2_I*_2_*(4,4′-bpy)(PR_3_)_2_]_x_ (4,4′-bpy = 4,4′-bipyridil) complexes [46], and a series of [Cu(NCS){(N)_2_ or (NN)}PR_3_], (N = pyridine, NN = 2,2′-bipyridil or 1,10-phenanthroline) complexes [47].

## 2. Synthesis and the Characteristics of Tris(aminomethyl)phosphines

**Figure 2:**
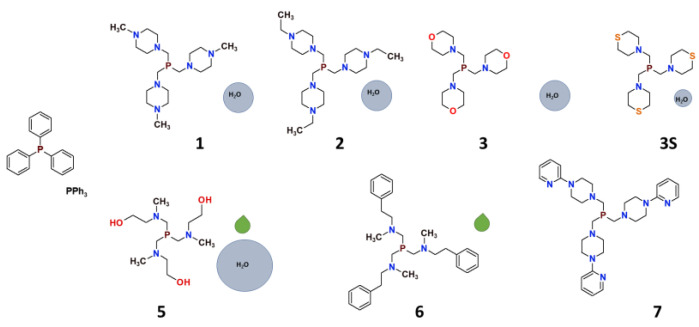
Tris(aminomethyl)phosphines reviewed in this work [8,11,13,16,17]. The size of the light-blue circles represents the relative water solubility of the compounds, and the green droplets represent compounds synthesized as viscous oils.

Syntheses of tris(aminomethyl)phosphines, conducted under anaerobic conditions, are straightforward and give high yields of the products. Phosphines chosen for this project (Figure 2) can be divided into two groups. The first one is composed of phosphines with aliphatic 6-membered ring substituents: the derivatives of *N*–methylpiperazine (tris((4-methylpiperazin-1-yl)methyl)phosphine; **1**) and *N*–ethylpiperazine (tris((4-methylpiperazin-1-yl)ethyl)phosphine; **2**) as well as morpholine (tris(morpholinomethyl)phosphine; **3**) [8,18] and thiomorpholine (tris(thiomorpholinomethyl)phosphine; **3S**) [13], and a bulky derivative of *N*–(2–pyridyl)piperazine (tris((4-(pyridin-2-yl)piperazin-1-yl)methyl)phosphine; **7**) [16]. The second group of the ligands consists of phosphines with branched substituents: the ligand synthesized originally by Krauter and Beller [48] derived from 2–aminomethyl–ethanol (2,2′,2′’-((phosphinetriyltris(methylene))tris(methylazanediyl))triethanol; **5**) [11] and the phosphine derived from *N*–methyl–2–phenyletanamine (*N*,*N*’,*N*’’-(phosphinetriyltris(methylene))tris(*N*-methyl-2-phenylethanamine); **6**) [17].

All the phosphines are well soluble in most common polar and nonpolar solvents. Some of them (**1**, **2**, **3,** and **5**) are also very well soluble in water. It should be highlighted that water or CHCl_3_ solutions of these ligands are air-stable at room temperature for a prolonged time, contrary to, for example, triphenylphosphine (PPh_3_), which readily undergoes oxidation in the air with the formation of triphenylphosphine oxide (O=PPh_3_). It should be noted that this feature turned out to be characteristic for all tris(aminomethyl)phosphines, and also for aminomethyldiphenylphophines.

**Figure 3:**
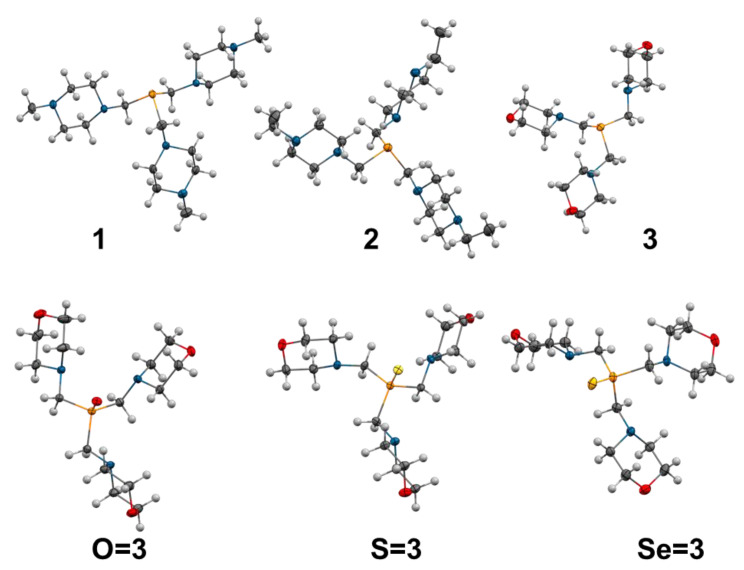
X-ray structures of the phosphines **1**: P(CH_2_N(CH_2_CH_2_)_2_NCH_3_)_3_ (CCDC 747837), **2**: P(CH_2_N(CH_2_CH_2_)_2_NCH_2_CH_3_)_3_ (CCDC 747841), and **3**: P(CH_2_N(CH_2_CH_2_)_2_O)_3_ (CCDC 747839) [8], and phosphine chalcogenides: **O=3** (CCDC 767991), **S=3** (CCDC 767992), and **Se=3** (CCDC 767993) [9]. Adapted with permission from ref. [8] (copyright 2011 Elsevier Inc.) and adapted from ref. [9] (copyright 2010 Royal Society of Chemistry).

**Figure 4:**
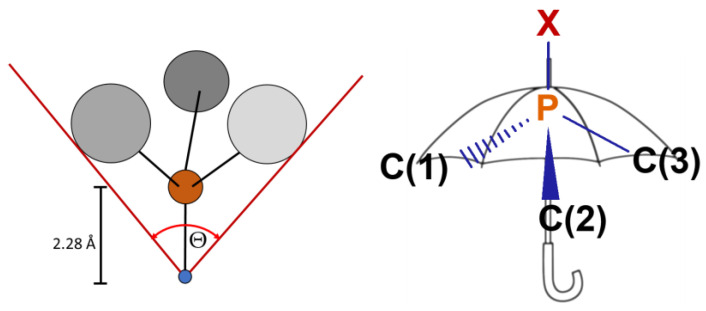
Definition of Tolman’s cone angles (θ) [49] and the symmetric deformation coordinate S4′—from the X-ray studies [50], S4—from the DFT calculations [51]. S4′(S4) = (XPC(1) + XPC(2) + XPC(3)) − (C(1)PC(2) + C(1)PC(3) + C(2)PC(3)).

The analysis of the structures obtained by X-ray crystallography (Figure 3) and DFT methods showed that the studied phosphines are characterized by the very large Tolman’s cone angles (θ, Figure 4 and Table 1) [9,49], defining steric hindrances of phosphine ligands. Notably, the θ values for the ring **1**, **2**, **3**, **3S**, and **7** are larger than for the branched **5** and **6**.

The S4′ parameter (the difference between the sum of angles XPC(1), XPC(2), and XPC(3) between the substituents and the coordinated metal atom, a chalcogenide atom at phosphorus atom, or electron lone pair (position determined using XPC(1) = XPC(2) = XPC(3) constrain) and the angles C(1)PC(2), C(1)PC(3), and C(2)PC(3) between substituents determined from the X-ray structures; Figure 4) [50] and the S4 parameter (the same, but from the DFT calculations) [51] are very similar for all the phosphines and adopt high values (Table 1). This shows that the tested ligands, despite the very high steric demands, are characterized by low steric hindrance in the immediate environment of the phosphorus atom.

The characteristics of electron-donating properties of the tested compounds were possible by using a subsequent parameter, which is the minimum of the molecular electrostatic potential (MEP) introduced by Koga and Suresh [53]. This parameter (V_min_) describes the changes in electronic density at the lone pair region of a substituted phosphine ligand as a result of the electron-donating or -withdrawing effect of the substituents. Its value indicates strong donor properties of **6** as well as **1** and **2** and weak donor properties of **3** and **3S**. V_min_ can be further extended into two factors: the steric effect (S_eff_) and the electronic effect (E_eff_) [54]. They quantify different contributions into the pure electron-donating properties of the lone electron pair of phosphines and other ligands. In general, the values of S_eff_ parameters for the studied phosphines are very similar, which was expected from the similar geometries around the phosphorus atom expressed as S4 parameter; however, the E_eff_ adopts very diversified values, demonstrating the ease of modifying the electron-donating properties of the phosphines by changing the substituents.

Studies on the chalcogenide derivatives (oxides, sulfides, and selenides) enabled a more comprehensive description of the physical and chemical properties of the studied phosphines. The X-ray structures of the oxide, sulfide, and selenide derivatives of **3** (**O=3**, **S=3** and **Se=3**, respectively; Figure 3) showed that the binding of the chalcogenide atom to the phosphine leads to significant changes in the geometry around the phosphorus atom: P–C bonds are shortened, and C–P–C bond angles are enlarged (Table 2). Binding the oxygen atom results in the biggest decrease in P–C bond lengths (from 1.845 to 1.821 Å) and the largest increase in C-P-C bond angles (from 98.8 to 103.5°). In the case of sulfide and selenide derivatives, these changes are slightly smaller and more random (1.822 Å and 103.4°, respectively, for **S=3**; 1.827 Å and 103.8°, respectively, for **Se** = **3**). Enlargement of the C–P–C angles results in drastic changes to the S4′ parameter. These values for chalcogenides are 34.5 for **O=3**; 35.1 for **S=3**; and 31.4 and 33.3 (av.: 32.4) for **Se=3**.

The structures determined with DFT methods (B3LYP/6-31G**) are close to the X-ray ones (sample data for **3** and its derivatives: Table 2), however one can observe some differences in the values of S4′ and S4 parameters, as well as in the P–C bond lengths, which are less dependent on the substituents in the case of calculated structures. This suggests that the geometries of the compounds are flexible and may depend on the organization of the molecules in the crystal net.

Analysis of the NMR spectra measured for all the phosphines and their derivatives (Table 2) confirms structural observations, indicating large changes in the electron distribution around the phosphorus atom upon formation of the bonds with chalcogenide atoms. Chemical shifts of the phosphorus atom in ^31^P{^1^H} NMR spectra for the free tris(aminomethyl)phosphines are −63–−59 ppm, depending on the phosphine. P–O(S,Se) bond formation significantly transfers the signals towards lower fields: by 106–107 ppm for the oxides and 104–105 and 88–89 ppm for the sulfides and selenides, respectively.

^1^H and ^13^C{^1^H} NMR spectra of the investigated phosphines and their derivatives reveal the averaged signals of all three substituents. This demonstrates that all these compounds in solution have symmetry C_3_ with freely rotating substituents. Moreover, the conformational changes of the aliphatic rings are relatively fast—the individual signals of axial and equatorial protons are observed only at low temperatures. Interestingly, the chemical shifts and coupling constants of H(1) and C(1) atoms from the –CH_2_– group directly linked to the phosphorus atom do not depend significantly on the type of substituent, but are very dependent on the type of the derivative, specially the ^1^J(PC^1^) values varying from ca. 4 Hz for the phosphines to ca. 80 Hz for the oxides.

In summary, all data showed a high impact of bonds formed by the phosphorus atom on the geometry around it, allowing one to extend the use of the S4 parameter (and to some extent the S4′ parameter) from a simple comparison of phosphine properties to the description of the changes of the surrounding of the phosphorus atom upon formation of the bond with a chalcogenide atom.

## 3. [CuX(NN)PR_3_] Complexes

Reactions of the phosphines with CuX (X = I or NCS) and diimine (NN) in a 1:1:1 molar ratio give complexes of the general formula [CuI(dmp)PR_3_] (Equation (1)):

CuX + NN + PR_3_→[CuX(NN)PR_3_] (1)

2[CuX(NN)PR_3_] = [Cu_2_X_2_(NN)_2_] + 2PR_3_ (2)

**Figure 5:**
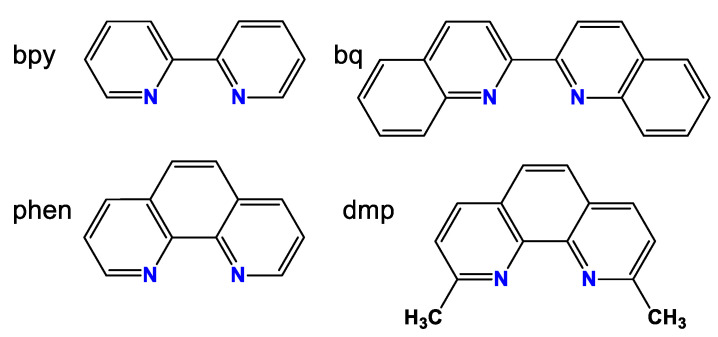
Schematic structures of 2,2′-bipyridyl (bpy), 1,10-phenanthroline (phen), 2,9-dimethyl-1,10-phenanthroline (dmp), and 2,2′-biquinoline (bq).

The first synthesized copper(I) complexes with tris(aminomethyl)phosphines were the CuI complexes with 1,10-phenanthroline (phen) or 2,2′-bipyridyl (bpy) (Figure 5) and **1**, **2** or **3** [8]: [CuI(bpy)**1**], [CuI(bpy)**2**], [CuI(bpy)**3**], [CuI(phen)**1**], [CuI(phen)**2**], and [CuI(phen)**3**]. Stability of the bpy complexes was rather small. The phen complexes were much more stable, but over time they underwent decomposition in the air and in the presence of water.

The next investigated group consisted of the CuI complexes with 2,9-dimethyl-1,10-phenanthroline (dmp) and **1**, **3**, PPh_3_ [10], and highly hydrophilic **5** [11]: [CuI(dmp)**1**], [CuI(dmp)**3**], [CuI(dmp)PPh_3_], and [CuI(dmp)**5**], which turned out to be insoluble in any of the solvents. Importantly, stability of the dmp complexes proved to be very high in air- and water-containing solutions—up to several weeks.

Due to a certain lability of the obtained compounds, a reaction of the phosphine dissociation followed by formation of the binuclear [Cu_2_X_2_(NN)_2_] species (eqn. 2) could not be excluded. Therefore, in some cases, the mixtures of the phosphines with CuX and NN in 2:1:1 molar ratios were also prepared, which resulted in the studies of the following systems: [CuI(phen)**1**]∙**1**, [CuI(phen)**3**]∙**3**, [CuI(dmp)**1**]∙**1**, [CuI(dmp)**3**]∙**3**, and [CuI(dmp)PPh_3_]∙PPh_3_ [10], as well as [CuI(phen)**5**]∙**5** and [CuI(dmp)**5**]∙**5** [11].

To extend the research on the copper(I) complexes with other anions than the iodide, we undertook the syntheses of the copper(I) thiocyanate complexes. The first one was [CuNCS(phen)**3**] [12]. In the next step, we synthesized CuNCS complexes with dmp and two structurally similar phosphines—the derivatives of morpholine (**3**) and thiomorpholine (**3S**) [13]. Three new complexes, [CuNCS(dmp)**3**], [CuNCS(dmp)**3S**], and the iodide [CuI(dmp)**3S**] were synthesized.

Finally, we studied the CuI and CuNCS complexes with more developed phosphines: the derivative of *N*–(2-pirydyl)piperazine (**7**) and the derivative of *N*–methyl-phenetylamine, the “branched” phosphine of lesser steric demands (**6**) [16,17]. Three new copper(I) iodide or isothiocyanate complexes with **6** and phen or dmp, [CuI(phen)6], [CuI(dmp)**6**], and [CuNCS(dmp)**6**] were synthesized, however it was not possible to obtain a pure, stable sample of [CuNCS(phen)**6**]. In the case of **7** only two complexes, [CuI(dmp)**7**] and [CuNCS(dmp)**7**], have been successfully synthesized.

In the last step, we decided to study the complexes with 2,2-biquinoline ligand (bq). This diimine has fused aromatic rings close to the nitrogen donor atoms (Figure 4), which results in considerable steric hindrances. In this context, bq is like dmp, and the synthesized [CuI(bq)**3**] complex, as expected, was as stable as the dmp ones, which was shown by measuring the UV-Vis spectra of [CuI(dmp)**3**] and [CuI(bq)**3**] for 48 h in air in the mixture of DMSO with 10% H_2_O [15].

The complexes were characterized using UV-Vis spectroscopy in solution and reflectance spectroscopy in solid state. In the case of isothiocyanate complexes, IR spectroscopy was also used. NMR spectroscopy was, however, the most useful technique, which allowed full characterization of the complexes in solutions. Table 2 presents selected NMR data for complexes of **3**. In all cases, formation of the [CuX(NN)PR_3_] complexes with tris(aminomethyl)phosphines resulted in a large shift of the phosphorus atom signal to the lower fields, from ca. −60 to ca. −30 ppm with a significant broadening of the signal. The spectra of mixtures of [CuX(NN)PR_3_]·PR_3_ showed two very broad signals at −30 and −60 ppm, what indicated the exchange of phosphine ligands and demonstrated the lability of the complexes. ^13^C{^1^H} and ^1^H NMR spectra confirm equally clearly the formation of new complexes. First, large changes were observed in the parts of the diimine ligands, but they remained symmetrical, indicating a tetrahedral structure typical for Cu(I) complexes. Second, the formation of complexes transformed also the phosphine parts of the spectra, especially the signals of H(1) and C(1) atoms of the -CH_2_- group directly linked to the phosphorus atom. As in the case of chalcogenides formation, proton signals drifted towards lower fields, and the signals of carbon atoms, towards higher fields. However, unlike for the chalcogenides, H(1) proton signals underwent broadening paired with the disappearance of the ^2^J(PH^1^) coupling constant. ^1^J(PC^1^) coupling constants grew to about 25 Hz for the phen and bpy complexes, while for the dmp and bq complexes, broadening of the signals and the disappearance of the coupling were observed.

### 3.1. X-ray Structures of the Complexes

**Figure 6:**
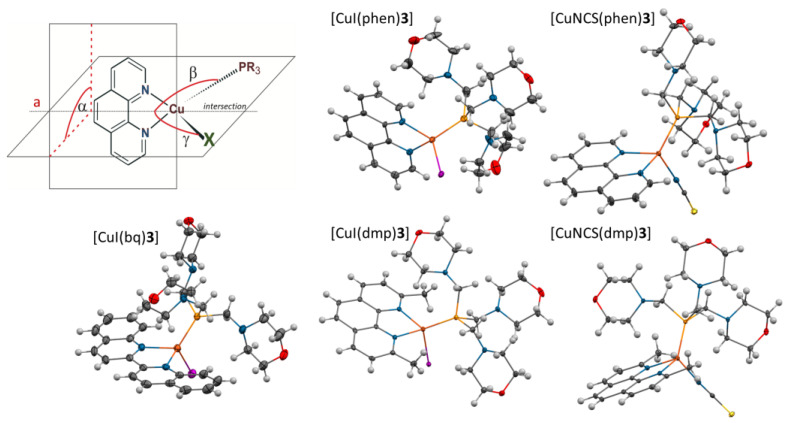
Structural distortions from a tetrahedral coordination sphere around a copper atom: α—the measure of the flattening distortions; | β − γ |—the rocking distortions [10]; and X-ray structures (25% thermal ellipsoids) of the complexes with **3** [8,10,13,15]. Adapted with permission from ref. [15] (copyright 2012 Elsevier Inc.) and adapted from refs. [8,10,13] (copyright 2010, 2011, 2013 Royal Society of Chemistry).

In the crystal structures of the investigated complexes, the central Cu(I) ion is surrounded by diimine ligand chelating by nitrogen atoms, thiocyanate ligand coordinating by nitrogen atom, or iodide ligand and a phosphine molecule, coordinating monodentally by phosphorus atom (Figure 6) [8,10,11,12,13,15,16,17]. All investigated complexes crystallize as discrete centrosymmetric dimers bound by π-stacking interactions between the diimine molecules, wherein a very wide range of various types of molecular overlap is observed. For the sake of further discussion, it should be mentioned that in the case of complexes [CuI(dmp)**3**], [CuNCS(dmp)**3**], and [CuI(dmp)**1**], there is only one type of interaction observed—with carbon atoms both overlapping others and placed in the centers of the stacked rings. For the complexes with **3S** ([CuI(dmp)**3S**] and [CuNCS(dmp)**3S**]), the diimine rings are additionally displaced in opposite directions.

The complexes are characterized by pseudotetrahedral geometry in which the deviations from the ideal tetrahedron result from the geometry imposed by the diimine and the difference between the (pseudo)halide ligand and phosphine molecule. They can be described by geometry-flattening distortions (Figure 6: α—dihedral angle between the diimine plane and the I-Cu-P plane) and rocking distortions (| β − γ |—the absolute value of the difference between the angles of the intersection of the aforementioned planes (a) and the Cu–P and Cu–I or Cu–NCS bonds, respectively).

Noticeable flattening distortions are observed for the phen complexes [8,11,17]—the value of the angle α oscillates in some cases as low as about 80° (Table 3). Moreover, coordination of the phosphine molecules leads to considerable change in their geometries. The values of the S4′ are reduced from ca. 60° to 50°. Furthermore, shortening of the P-C bonds is observed in the case of the complexes with **1** and **3**. These effects are consistent with those observed for chalcogenide derivatives, although the changes are much smaller. In addition, they are consistent with the ^13^C{^1^H} NMR spectra, in which the signals of the C(1) carbon atoms are significantly shifted toward higher fields and coupling constants ^1^J(PC(1)) equal to 25 Hz (Table 2) are smaller than constants observed for chalcogenides, but remain much larger than observed for the free phosphines.

For the dmp complexes, the values of the α angles are close to 90°, indicating small flattening distortions. Bond lengths around the copper atom do not differ significantly from those in the phen complexes [8,10,11,12,13,16,17]. The formation of the complexes obviously generates changes in the geometries of the phosphine ligands, which are, however, different than those for the phen ones. While one can observe significant shortening of the P–C bonds for phen complexes, a slight lengthening is observed in the dmp ones (Table 3). Simultaneously, the S4′ values are considerably bigger in the dmp complexes, close to the values for the free phosphines. The differences between the complexes with phen and dmp, observed also in the NMR spectra, certainly result from the presence of the methyl substituents in the dmp ligand, which considerably limit the available space around the copper central atom. This results in atypical changes of the phosphine geometries and in a significant prolongation of the bond between the copper and iodide anion characterized by a large ionic radius (2.06 Å [55]).

Interestingly, the steric constraints generated by the methyl groups in the dmp ligand are not observed in case of the bq complexes [15], despite the fact that bq should be characterized with approximately equal requirements. Cu–P bond length in [CuI(bq)**3**] (Figure 3, Table 3) is longer only by 0.01 Å in comparison to [CuI(dmp)**3**], however, the average P-C(1) bond length is comparable to bond lengths found in the phen complexes. The S4′ value for **3** in the studied complex (44.7) is significantly smaller than the value not only for **3** in [CuI(dmp)**3**] (58.1), but also for **3** in [CuI(phen)**3**]. Moreover, Cu–I is the shortest one among the investigated complexes. Most probably tis results from the specific structure of bq, which is not planar in the complex; the torsional angle between the planes of the fused rings is equal to 10°.

Structural studies of complexes with DFT methods allowed the analysis of the isolated molecules, as for the chalcogenide derivatives of tris(aminomethyl)phosphines. The calculations showed that the geometry of the ground singlet states differs slightly from the geometry of the X-ray structures. Calculated S4 parameters are close to the crystallographic parameters S4′, however they are slightly lower (Table 2). The calculations gave also longer bonds of phosphorus with C(1) carbon atoms of the substituents compared to the crystallographic data, but it should be emphasized that they reproduced the observed relationship: P–C(1) bond lengths in complexes with phen and bq are shortened in relation to the bonds in the free phosphines, although not as much as for the chalcogenide derivatives; in complexes with dmp, slightly longer. The values of the α angle defining the geometry-flattening in most cases are closer to 90°, which seems to be reasonable for the isolated molecules.

### 3.2. Solid-State Luminescence

All complexes, except the bq ones, exhibit photoluminescence in solid state [8,10,11,12,13,16,17]; however, its intensity was found to be weak in the case of the phen complexes or very weak in the case of the bpy ones [8,12,17]. The dmp complexes proved to be much better phosphors. Their emission can be seen with the naked eye, which is reflected in their relatively long luminescence lifetimes. It should also be noted that none of the complexes showed photoluminescence properties in solutions.

Mechanisms responsible for the photophysical properties of the copper(I) complexes are well understood [33,34,35,36,37,38,39,40,41,42,43,44]. Upon excitation, these complexes undergo the metal-to-ligand-charge-transfer (MLCT) transition where one electron is transferred from the Cu(I) center to a diimine ligand, forming a transient Cu(II) species. The resulting MLCT state undergoes a significant structural reorganization because of its transformation from the closed-shell 3d^10^ in the ground state to an open-shell 3d^9^ electron configuration, which leads to flattening of the pseudotetrahedral geometry. In a typical UV-Vis spectrum there are two bands, S^0^ → S^2^ and S^0^ → S^1^, with an ultrafast internal conversion of S^2^ to S^1^ state. Next, the singlet MLCT state undergoes an intersystem crossing to produce an emissive triplet MLCT state [33,34,35,36,37,38,39,40,41,42,43,44].

For the complexes discussed herein, the same phenomenon is observed. Their emission spectra are characterized by one broad band. The only exception was the laser-induced spectrum of irregular shape recorded for [CuNCS(phen)**3**] [11], indicating a multicomponent character of its emission. Excitation spectra measured for the selected complexes [10] corresponding to the reflectance spectra and double luminescence decay times confirmed mechanisms typical for the Cu(I) complexes.

For all studied complexes, changing the temperature from 298 to 77 K causes a typical decrease in the luminescence intensity, accompanied by a bathochromic shift and a significant decrease in the band half-width, which is also typical for copper(I) complexes [33,34,35,36,37,38,39,40,41,42,43,44]. Regardless of the excitation wavelength, the maxima of the emission bands for most dmp complexes are located at ca. 600 nm at room temperature and are significantly shifted towards the shorter wavelengths as compared with complexes with bpy and phen, for which the maxima are placed at ca. 650 nm.

Analysis of the influence of (pseudo)halide anion on the luminescence spectra of the dmp complexes shows that the bands of the thiocyanate complexes are much wider than the bands observed for the iodide ones, whereas the luminescence lifetimes are several times shorter. In some cases, the positions of the band maxima depend on the coordinated anion (CuI and CuNCS complexes with dmp and **6** or **7**), which is in agreement with the literature data [56,57].

Interestingly, in two cases the maxima of the luminescence band considerably depend on the phosphine used (**3** or **3S**), and the spectra of complexes with **3S** are strongly shifted to lower energies [13]. Although estimated electronic parameters for **3S** differ noticeably from those for **3**, this was unexpected, since the luminescence maxima in the spectra of the CuI complexes with **1**, **3**, and PPh_3_ are virtually the same [10]. These apparent discrepancies can be explained by analyzing the arrangement of molecules in the crystal lattice. The lack of dependence of the luminescence maximum on the phosphine ligand found for [CuI(dmp)**1**], [CuI(dmp)**3**], and [CuI(dmp)PPh_3_], characterized with the same π-stacking pattern, allowed us to conclude that these interactions are most likely responsible for the emission maximum wavelength, and the role of the phosphine ligands and (pseudo)halogen anions consists rather in the modifications of the crystal cell packing [10,13].

TDDFT calculations showed that for all the complexes, regardless of the diimine, phosphine, and anion type, very similar transitions are observed. For all investigated complexes there are two intense transitions corresponding to the maximum of the CT band, which can be ascribed as (MX,MPR_3_)LCT with electron transfer from the Cu–I or Cu–NCS bond with a small admixture of the Cu–P bond to π* orbitals of the phen or dmp ligand. The first triplet transitions (T1), corresponding to the emission processes, for all the compounds, similar to S1, are of the (MX)LCT type [10,13].

Even though significant changes in the geometry of the molecules in solid state are of course not possible, studies using time-resolved measurements of the X-ray structure of the [Cu(dmp)(Ph_2_PCH_2_CH_2_PPh_2_)]^+^ complex revealed slight structural changes in the molecules in the crystal lattice upon excitation [33]. This effect was reflected in the studies of the ultrafast excited-state dynamics of the [Cu(dmp)_2_]^+^ complex investigated using the on-the-fly surface-hopping approach [58] and also in our DFT calculations, in which the magnitude of the molecular deformations in the triplet state correlate well with the observed differences in the luminescence decay times for different groups of compounds and luminescence intensity. Optimization of the triplet state may be therefore very useful in predicting the luminescent characteristics of the complexes of copper (I), even in solid state.

## 4. Antimicrobial Activity

The studied phosphines and complexes were screened for their in vitro antibacterial activity against Gram-negative *Escherichia coli* PCM 2057 (ATCC 25922) and *Pseudomonas aeruginosa* (clinical sample), as well as Gram-positive *Staphylococcus aureus* PCM 2054 (ATCC 25923), and for in vitro antifungal activity against *Candida albicans*. (clinical sample). The minimal inhibitory concentrations (MIC) are collected in Table 4.

Uncoordinated phosphines were inactive against each of the investigated microorganisms. Activity of the bpy complexes was slightly higher, and the complexes with phen showed noticeable activity, especially towards *S. aureus*. Interestingly, the activity of [CuI(phen)**1**]·**1** and [CuI(phen)**3**]·**3** mixtures was higher than the activity of pure [CuI(phen)**1**] and [CuI(phen)**3**] complexes, despite the fact that phosphines **1** and **3** were inactive. This can only be explained by the relatively low stability of [CuI(phen)**1**] and [CuI(phen)**3**] complexes resulting from the dissociation of the phosphine ligand.

The dmp complexes are characterized by a significant antibacterial activity, and *S. aureus* was the most susceptible to the tested complexes; activity against this bacterium was comparable to the activity of gentamicin (1–5 μg/mL). High antifungal activity against *C. albicans* of the dmp complexes should also be underlined. Antimicrobial activity of [CuI(bq)**3**] can be located between the activity of [CuI(dmp)**3**] and [CuI(phen)**3**], which confirms a high impact of diimine ligands on the properties of the studied complexes.

Even though the phosphine used does not significantly affect the activity of the complexes, a high increase in the activity of the dmp and phen complexes with the strongly hydrophilic phosphine **5** was observed.

## 5. In Vitro Cytotoxicity

For the initial studies on the in vitro antitumor activity of the iodide complexes with **1**, **3**, or **5** and with phen or dmp, two human ovarian carcinoma cell lines were selected: a line resistant to cisplatin (SKOV 3) and a sensitive one (MDAH 2774) [11]. The tests were conducted only for the complexes with one additional molecule of the phosphine. Results show a strong cytotoxic effect (expressed as IC_50_; Table 5) of the complexes on both cell lines, much stronger than of cisplatin used as a reference.

Regarding MDAH 2774 cells, the activity of the studied complexes was lower, but more diversified [11]. It should also be noted that the activity of the investigated complexes was remarkably higher than the activity of pure ligands, both the diimines and the phosphines (500–100 μM against both lines).

Tests of the iodido and thiocyanato complexes with dmp and phosphines **3** and **3S** (Table 6) were performed using two cell lines: mouse colon carcinoma (CT26) and human lung adenocarcinoma (A549). The study revealed that, also in this case, the tested Cu(I) complexes were much more active than cisplatin. Moreover, it was observed that the A459 line generally was more sensitive to the tested compounds. This means that the studied complexes may exhibit selective biological activity only towards specific tumor cell lines. Furthermore, the cytotoxic activity after 24 and 48 h of cell incubation with the studied compounds was analyzed. Extended incubation time resulted in much higher concentrations needed to reduce the surviving fraction by 50%. Taking into consideration the time of cell division (A549: 24 h; CT26: 48 h) the obtained results seem to be within reason, and much lower IC_50_ values for 48 h most probably are related to the cell-line ageing.

The cytotoxic effect (degenerative changes in cells) of the phosphines **1**, **2**, and **3** [9], as well as complexes [CuI(dmp)**3**] and [CuI(bq)**3**] [15], was tested additionally on the non-tumor cell lines. Phosphines, considered commonly as a very toxic class of compounds, proved to be inactive against the sensitive continuous cell line Vero (derived from kidney epithelial cells of the African green monkey *Cercopithecus aethiops*) in concentrations up to 16 mM. The cytotoxic effect of the complexes was tested on the cell line RK-13 (rabbit kidney) and mesenchymal stem cells (MSC) isolated from horse adipose tissue. In the case of RK-13, the cytotoxic effect appeared after 24 h and remained throughout the incubation time. [CuI(bq)**3**] was cytotoxic in concentrations up to 1 × 10^−2.5^ µM, whereas [CuI(dmp)**3**] was cytotoxic in concentrations up to 1 × 10^−4^ µM. In the case of MSC cells, high inhibition of metabolism was noticed at all tested concentrations (200–0.1 μM) for [CuI(dmp)**3**]. The activity of [CuI(bq)**3**] was much lower (IC_50_ ~ 2 μM).

## 6. Interactions with Plasmid DNA and Serum Albumins

It is widely recognized that studies of interactions between potential drugs and biomolecules are an important part of the evaluation of their potential. The most popular biotargets are still DNA and transporting serum albumins.

**Figure 7:**
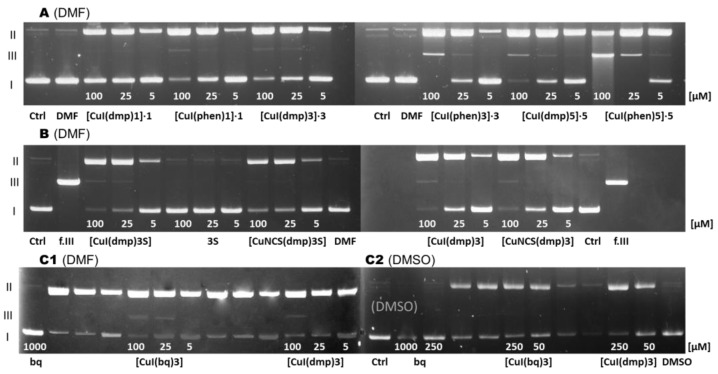
Plasmid DNA cleavage (in the 10% DMF buffered solutions: (**A**) [11], (**B**) [14], (**C1**) [15]; in the 10% DMSO buffered solutions: (**C2**) [15]).

Impact of the studied compounds on plasmid DNA was tested using electrophoresis (Figure 7). Plasmid naturally occurs as a covalently closed superhelical form (form I), which is effectively separated from relaxed/nicked plasmid (form II) and linear DNA (form III) formed as a result of single- or double-strand breaks.

Since the complexes are hardly soluble in water, they were predissolved in dimethyl formamide (DMF). Initial studies of the cleavage activity of **3** and its oxide, sulfide, and selenide derivatives [9] showed that these compounds, even in very high concentrations (2000 μM), caused formation only of small amounts of form II. **3S** did not cause DNA damage in the tested concentrations (≤100 μM) [14]. In addition, the dmp and bq ligands did not cause any DNA lesions (≤1000 μM). This corroborates the low antimicrobial and cytotoxic activity of the diimines and the phosphine ligands.

The first tested group of complexes consisted of the complexes of CuI with phen and dmp and phosphines **1**, **3**, or **5** [11]. The ability of the complexes to induce single- and double-strand breaks formed the following order: [CuI(dmp)**1**]∙**1** < [CuI(dmp)**3**]∙**3** ≈ [CuI(phen)**1**]∙**1** < [CuI(dmp)**5**]∙**5** < [CuI(phen)**3**]∙**3** << [CuI(phen)**5**]∙**5** (Figure 7A). This clearly showed that the dmp complexes were less efficient in causing DNA damage than the phen ones, what is not surprising, considering the strong intercalating potential of the phen ligand and its ability to modulate the redox activity of metal complexes [59]. For both classes, the complexes with phosphine **1** were the least efficient. Their presence only caused a single strand break. The complexes with phosphine **3** caused greater damage to the plasmid. The efficiency of DNA degradation is the most intensive in the complexes with **5**. At a concentration of 100 μM, the [CuI(phen)**5**] **5** complex was found to provoke the formation of the linear form (III); in addition, some amounts of unidentified species, seen as continuous luminescent trace, were observed.

In the second study [14], the electropherograms were made for [CuI(dmp)**3**] and [CuI(dmp)**3S**] as well as [CuNCS(dmp)**3**] and [CuNCS(dmp)**3S**]. Results indicate that all the complexes are comparably efficient in the DNA degradation process; however, for the iodido ones, the degradation process is slightly more intensive. The ability of the complexes to induce single- and double-strand breaks can be ordered as follows: [CuNCS(dmp)**3S**] < [CuNCS(dmp)**3**] ≤ [CuI(dmp)**3**] < [CuI(dmp)**3S**] (Figure 7B).

Finally, the ability of the [CuI(bq)**3**] and [CuI(dmp)**3**] complexes to induce DNA lesions was monitored in buffered 10% solutions of DMSO and DMF [15] (Figure 7(C1,C2)). DMSO was used additionally as a solvent because it is an effective radical scavenger [60]. Indeed, in DMSO, even high concentrations of both investigated compounds only cause a single strand break. In DMF, on the other hand, the [CuI(dmp)**3**] complex causes the formation of form II and certain amounts of form III at a concentration of 50 μM, and [CuI(bq)**3**] forms form III at 50 and 25 μM. This shows that [CuI(bq)**3**] is more efficient in DNA degradation. On the other hand, prevention of double lesions by DMSO suggests that a free radical mechanism of action on the plasmid DNA degradation process is involved.

To summarize, the activity of the phen complexes, and surprisingly of the bq ones, is higher than the activity of the dmp ones, which are characterized by the highest antimicrobial activity. Moreover, differences in the abilities to induce DNA lesions do not reflect observed differences in the cytotoxicity in vitro against different cell lines. This suggests that the mechanisms of their biological activity are not associated with DNA damage. It is worth noting that remarkably low activity of the ligands towards DNA makes the phosphines and the diimines safe choices as parts of potential metal-based drugs.

Interactions with bovine serum albumin (BSA) and human serum albumin (HSA) are commonly investigated because serum albumins not only are the key proteins for the transport of drugs in the blood plasma, but also have often been used as a substitute for a “typical” protein during numerous biophysical, biochemical, and physicochemical studies. The measurement of albumin fluorescence quenching is the most important method of investigating the interactions of drugs with serum albumins, due to the highly luminescent tryptophan residues, which can be easily quenched upon interactions with different compounds. BSA has two tryptophan residues: Trp-212, located within a hydrophobic binding pocket of the protein, and Trp-134, placed on the surface of the molecule. However, HSA has only one (Trp-214) in the hydrophobic pocket.

For these studies, we used a non-typical approach, namely, the complexes were prepared as thin films on the walls of the tubes by a fast evaporation of a CHCl_3_ solvent in a strong stream of dinitrogen. Then they were incubated with albumins for at least 0.5 h at room temperature. During that time, complexes were partially dissolved in the solution (in the two lowest concentrations, the complexes were dissolved completely). Since the complexes are not soluble in water or buffers, the amount of the compound dissolved in the presence of HSA or BSA was an indirect indicator of the affinity of the compounds to the albumins.

As in the case of plasmid DNA cleavage, three different experiments were performed. The first group of complexes [11] was tested towards BSA. Ability of tryptophan quenching in BSA formed the following order: [CuI(dmp)**5**]∙**5** ≤ [CuI(dmp)**3**]∙**3** ≤ [CuI(dmp)**1**]∙**1** ≤ [CuI(phen)**1**]∙**1** < [CuI(phen)**3**]∙**3** << [CuI(phen)**5**]∙**5**; this shows that phen complexes generally seem to bind BSA more strongly than the dmp ones. It should be noted that the progress of the Stern–Volmer curves does not reveal a linear correlation between (I_0_/I-1) and the concentration of the complexes. The positive deviations from the slope at higher concentrations, together with a small influence of the complexes on the ternary structure of the albumin (measured as the Cotton effect using the circular dichroism spectroscopy), indicate a mixed mechanism of quenching of the surface Trp-134 residue, rather than involving the quenching of Trp-212 placed in the hydrophobic pocket. For the second group of complexes, [CuI(dmp)**3**], [CuNCS(dmp)**3**], [CuI(dmp)**3S**], and [CuNCS(dmp)**3S**], the interactions were tested for both HSA and BSA albumins [14]. Apart from a high binding ability of [CuI(dmp)**3**], both complexes with **3** quenched the luminescence of BSA more strongly, whereas the complexes with **3S** quenched the luminescence of HSA. Taking into consideration that BSA luminescence quenching caused by [CuI(dmp)**3**] was the weakest within other groups of complexes, the obtained results suggest that all four complexes do not strongly interact with both albumins. Analysis of the tryptophan quenching by the last set of complexes ([CuI(bq)**3**] and [CuI(dmp)**3**]) [15] showed that both compounds more strongly quench the BSA luminescence and the [CuI(dmp)**3**] complex is a much more efficient quencher for both albumins than [CuI(bq)**3**]. In order to verify the binding site, a site I marker (warfarin) was used. It forms a highly luminescent complex with these two albumins. The measurements of quenching effects, resulting from the substitution of the warfarin with [CuI(dmp)**3**] or [CuI(bq)**3**], showed a certain reduction of the intensity of luminescence of the warfarin–albumin complex in the presence of the investigated complexes, suggesting that both compounds bind to HSA and BSA, similar to warfarin, within the subdomain IIA in SSI (Sudlow’s site I) [61].

Admittedly, these experiments, due to unconventional preparation of the mixtures, could not bring quantitative results. However, additional measurements of the absorption spectra of the investigated systems showed that in all cases, albumins interacted with the stable Cu(I) complexes and no copper oxidation occurred, what was proved by the presence of the characteristic MLCT bands in the visible region of the UV-Vis spectra.

## 7. Conclusions

Despite the fact that tris(aminomethyl)phosphines have been known since the 1960s, the complexes presented herein belong to a group of the first transition metal complexes carrying this type of ligand. The spectroscopic, structural, theoretical, and biological studies in this project allowed us to draw several general conclusions:

(1) Tris(aminomethyl)phosphines are characterized by extremely low antimicrobial activity, negligible in vitro cytotoxicity, and low activity in inducing DNA damage. This finding is particularly important, because phosphines are commonly considered to be a toxic class of compounds. There is a strong certainty among the community of chemists that phosphines belong to a very poisonous class of compounds, which probably originates from a high toxicity of phosphorus trihydride PH_3_ [62,63]. Sometimes it is connected to the fact that some of the molecules of the chemical warfare gases (Tabun, Sarin, Soman, VX [64]) contain phosphorus atoms. Admittedly, when starting this project, we expected a high biological activity of these ligands; however, our studies showed that phosphines, at least tris(aminomethyl)phosphines, were not active in most of the in vitro tests.
(2) Regarding the structural aspects, the presented phosphines are characterized by very large Tolman’s cone angles, which indicates their high steric demands. On the other hand, the structural S4′ and S4 parameters of the tris(aminomethyl) phosphines are also high, which indicates a low bulkiness of these compounds in the direct vicinity of the phosphorus atom and makes them convenient ligands in the synthesis of transition metal complexes. It should be noted that tris(aminomethyl)phosphines may be a good alternative for commonly used triphenylphosphine (PPh_3_) in the complexes of different transition metals. A great example of successful usage of a less popular phosphine ligand are two ruthenium(II) complexes, RAPTA-C and RAPTA-T [2], bearing a molecule of 1,3,5-triaza-7-phosphaadamantane, which also can be regarded as a tris(aminomethyl)phosphine.
(3) Stability of the Cu(I) (pseudo)halogenide complexes with phosphines and diimines depends mainly on the diimine ligand. Complexes with 2,2 ‘-bipyridyl (bpy) are the least stable, complexes with 1,10-phenanthroline (phen) proved to be much more stable, and the stability of complexes of 2,9-dimethyl-1,10-phenanthroline (dmp) and 2,2 ‘-quinoline (bq) ligands is very high if not unlimited.
(4) TDDFT theoretical studies showed that MLCT absorption and emission bands observed for the studied complexes may be more accurately defined as (MX,MPR_3_)LCT bands resulting from the electron transfer from the copper–(pseudo)halide bond with a small admixture of copper–phosphorus bond to the π* orbitals of diimine ligands.
(5) Significant differences in the position of the emission bands maxima observed for the complexes with various anions and phosphine ligands have led us to a rather unexpected conclusion. Namely, the position of the luminescence bands depends mostly on the orientation of molecules in the unit cell, which directly affects the type of the π-stacking interactions between the diimine rings. The role of the other ligands is reduced to modifications of the molecular packing in a crystal cell.
(6) The complexes with highly sterically demanding dmp are characterized by high solid-state photoluminescence intensity and relatively long lifetimes in contrast to complexes with bpy and phen. The luminescence intensity also depends on other ligands and is higher for the iodide complexes than the thiocyanate ones, what is reflected in the significantly shorter luminescence lifetimes of the latter complexes. Structural analysis with DFT methods showed that the main factor determining the luminescence intensity is the rigidity of the immediate surroundings of the copper ion. For complexes with the ligands with high steric requirements, the possibility of deformation of the molecule in an excited state is minimal, therefore the probability of nonradiative transfer to the ground state is also much lower, resulting in elongation of the MLCT excited state lifetimes and more intense luminescence.
(7) The copper complexes presented herein are interesting as potential antibacterial and antifungal agents, as well as potential cytostatics, as demonstrated by the in vitro studies on selected cell lines. A relatively high selectivity of cytostatic (or cytotoxic) properties makes these compounds promising potential therapeutics.
(8) The biological properties of the complexes are most probably a function of the mixed molecular structure. On one hand, we have a diimine molecule capable of π-stacking interactions with rings of tryptophan, tyrosine, and phenylalanine and partial intercalation to the DNA or RNA chain; on the other hand, we have a phosphine ligand with easily modifiable steric properties, hydrophilicity, and a capacity of potential specific interactions. The type of the (pseudo)halide and phosphine used affects the biological activity of the complexes much less, but it is not negligible.
(9) Complexes with dmp showed the greatest activity. The complexes with bq and phen were less active, although in the latter case this is most likely caused by the relatively low stability.
(10) The interactions between the tested complexes and biomolecules confirm the promising properties of these compounds. Importantly, they bind to serum albumins without changing their tertiary structure and without oxidation and decomposition. Most of the complexes induce DNA degradation through a free radical mechanism; however, they cause a double cut of plasmids only at high concentrations.

Undoubtedly, studies of tris(aminomethyl)phosphines and their complexes presented herein are fundamental. Nevertheless, these complexes, regarded as simple models, molded a platform for further studies of more advanced systems. Indeed, the exploration of luminescence properties of the discussed complexes leaves space for improvement. Four diimine ligands were used, and obtained results clearly showed that the project could include studies of the complexes with different aromatic diimines providing even better emitting properties [65]. This is especially important in the context of the studies of the aggregation-induced luminescence phenomenon, which is gaining almost exponentially growing interest in the biomedicine area as a new way for cell imaging [66,67,68]. It should be noted that in the discussed project, diimine ligands combined with (pseudo)halides were used mainly to stabilize the resulting copper(I) complexes and to avoid dissociation of the complexes and the release of Cu(I) in the cells, which was observed for homoleptic Cu(I) complexes with monodentate phosphines [7,24]. The potential of the diimine ligands resulting from their redox properties [69] and their impact on the biological activity of the complexes was not thoroughly explored.

Hopefully, this review will draw more attention to this class of compounds, providing background for in-depth studies of this fascinating class of Cu(I) complexes.

## Figures and Tables

**Table 1:** Steric and electronic parameters of the investigated phosphines.

	1	2	3	3S	5	6	7	PPh_3_
XRD								
av. d(P-C) [Å]	1.855	1.856	1.845					1.830 [52]
av. α(C-P-C) [deg]	97.89	97.91	98.8					102.7 [52]
S4′	64.8	64.6	59.8					8.5 [52]
DFT								
Θ [deg]	210.2	218.9	206.7	213.5	194.8	194.7	207.7	145
S4	63.7	63.7	63.4	63.5	63.4	63.2	64.2	40.2
V_min_ [kcal/mol]	−35.74	−36.06	−30.38	−27.95	−31.71	−35.10	−32.22	−34.67
S_eff_	2.70	2.73	2.66	2.74	2.74	2.67	2.67	6.41
E_eff_	4.92	5.21	−0.40	−2.91	0.85	4.51	1.43	0.15

S4 = ΣXPC(i) − ΣC(i)PC(i); Θ—Tolman’s cone angle; V_min_—molecular electrostatic potential minimum (MEP); and S_eff_ and E_eff_—steric effect and electronic effect, respectively, in MEP.

**Table 2:** Selected data from ^31^P{^1^H}, ^13^C{^1^H}, and ^1^H NMR spectra (298 K, δ (ppm), J (Hz), in acetone-*d_6_*—organic molecules and CDCl_3_–Cu(I) complexes) and structural parameters of **3**, its chalcogenide derivatives, and **3** in Cu(I) complexes from the X-ray and DFT structures.

Compound	Ref.	NMR					X-ray		DFT	
		δ(P)	δ(C^1^)	^1^J(PC^1^)	δ(H^1^)	^2^J(PH^1^)	S4′	Average r(P–C)	S4	Average r(P–C^1^)
**3**	[8,9]	−62.8	59.3	4.3	2.64	2.9	59.8	1.845(3)	63.4	1.8820
**O = 3**	[9]	44.2	55.0	81.2	2.82	7.2	34.5	1.821(2)	32.5	1.8532
**S = 3**	[9]	42.4	58.0	67.0	3.03	5.2	35.1	1.822(2)	39.3	1.8694
**Se = 3**	[9]	26.3	57.8	59.8	3.18	4.6	31.4	1.825(3)	39.9	1.8688
							33.3	1.829(3)		
[CuI(bpy)**3**]	[8]	−35 *	55.8	26.0	2.88 *	-				
[CuI(phen)**3**]	[8]	−35 *	56.4	24.4	2.87 *	-	50.7	1.837(6)	49.6	1.8750
[CuNCS(phen)**3**]	[12]	−32 *	55.5	25.9	2.76 *	-	53.7	1.843(1)	48.8	
[CuI(dmp)**3**]**3**	[10]	−29 *; −60 *	56.6 *	-	2.77 *	-				
[CuI(dmp)**3**]	[10,13]	−28 *	55.8 *	-	2.88 *	-	58.1	1.855(2)	57.6	1.8844
[CuNCS(dmp)**3**]	[13]	−30 *	56.5 *	-	2.74 *	-	62.0	1.853(2)	56.5	1.8819
[CuI(bq)**3**]	[15]	−28 *	55.2 *	-	2.78 *	-	44.0	1.844(3)	54.1	1.8421

*—significantly broadened signal; δ—chemical shift (ppm; J—coupling constant (Hz).

**Table 3:** Selected X-ray structural parameters for the complexes with **3**. Adapted with permission from refs. [12,15] (copyright 2012 Elsevier Inc.) and adapted from refs. [8,10,13] (copyright 2010, 2012 Royal Society of Chemistry).

Ref.	Compound	Cu1–I1	Cu1–N1	Cu1–N1–C1	Cu1–P1	Average (P1–C)	*α*	| *β − γ* |	S4′
[8]	**3**					1.845(3)			59.8
[8]	[CuI(phen)**3**]	2.631(1)			2.193(2)	1.837(6)	79.8	12.0	50.7
[12]	[CuNCS(phen)**3**]		1.989(1)	164.81(9)	2.1885(3)	1.843(1)	89.2	10.2	53.7
[10]	[CuI(dmp)**3**]	2.674(1)			2.206(1)	1.855(2)	85.6	33.3	58.1
[13]	[CuNCS(dmp)**3**]		1.969(1)	175.27(12)	2.197(1)	1.853(2)	89.2	20.2	62.0
[15]	[CuI(bq)**3**]	2.609(2)			2.216(2)	1.844(3)	80.1	13.8	44.7

**Table 4:** Antibacterial and antifungal properties of the studied complexes (MIC [µg/mL]) against *E. coli*, *P. aeruginosa*, *S. aureus* and *C. albicans*. Adapted with permission from ref. [11] (copyright 2011 Elsevier Inc.), Adapted with permission from ref. [14] (copyright 2013 John Wiley & Sons A/S.) and adapted from ref. [8] (copyright 2010 Royal Society of Chemistry).

Ref.	Compound	*E. coli*	*P. aeruginosa*	*S. aureus*	*C. albicans*
[8]	**1**	>2560	>2560	>2560	640
	**2**	>2560	>2560	>2560	>2560
	**3**	1280	1280	640	1280
	[CuI(bpy)**1**]	2560	2560	320	1280
	[CuI(bpy)**2**]	1280	2560	320	2560
	[CuI(bpy)**3**]	640	2560	320	2560
	[CuI(phen)**1**]	320	2560	80	160
	[CuI(phen)**2**]	320	1280	80	160
	[CuI(phen)**3**]	320	2560	80	160
[11]	[CuI(phen)**1**]·**1**	160	1280	40	160
	[CuI(phen)**3**]·**3**	160	2560	20	160
	[CuI(phen)**5**]·**5**	160	*	20	40
	[CuI(dmp)**1**]	80	*	5.0	2.5
	[CuI(dmp)**1**]·**1**	80	2560	2.5	2.5
	[CuI(dmp)**3**]	80	*	2.5	2.5
	[CuI(dmp)**3**]·**3**	80	2560	2.5	2.5
	[CuI(dmp)**5**]·**5**	80	*	2.5	1.25
[14]	[CuNCS(dmp)**3**]	250	*	2	1
	[CuI(dmp)**3S**]	200	*	2	1
	[CuNCS(dmp)**3S**]	250	*	2	2
	ciprofloxacin	0.1	*	0.5	>300
	gentamycin	10	*	5	>300
	ampicillin	1	*	0.2	>300
[15]	[CuI(bq)**3**]	>300	*	20	100

*—not tested.

**Table 5:** IC_50_ values [μM] for two ovarian cancer cell lines (resistant-to-cisplatin SKOV 3 and cisplatin-sensitive MDAH 2774) following exposure to the complexes for 24 h based on the dose–response curves as derived from the MTT assay. Adapted with permission from ref. [11] (copyright 2011 Elsevier Inc.).

Ref.	Compound	SKOV 3	MDAH 2774
[11]	[CuI(phen)**1**]·**1**	3.2 ± 0.3	7.0 ± 0.7
	[CuI(phen)**3**]·**3**	1.9 ± 0.1	6.5 ± 0.2
	[CuI(phen)**5**]·**5**	2.2 ± 0.3	4.2 ± 0.2
	[CuI(dmp)**1**]·**1**	1.8 ± 0.1	2.0 ± 0.5
	[CuI(dmp)**3**]·**3**	2.2 ± 0.1	3.0 ± 0.1
	[CuI(dmp)**5**]·**5**	2.0 ± 0.4	4.0 ± 0.4
	cisplatin	180.5 ± 9.3	77.2 ± 7.6

**Table 6:** IC_50_ values [µM] obtained for CT26 (mouse colon carcinoma) and A549 (human lung adenocarcinoma) cell lines after 4, 24, and 48 h incubation time. Adapted with permission from ref. [14] (copyright 2013 John Wiley & Sons A/S.).

		4 h		24 h		48 h	
Ref.	Compound	CT26	A549	CT26	A549	CT26	A549
[14]	[CuI(dmp)**3**]	9.06 ± 0.48	1.56 ± 0.39	14,650 ± 340	280 ± 60	193.53 ± 11.31	35.03 ± 6.26
	[CuNCS(dmp)**3**]	6.78 ± 0.47	4.99 ± 0.40	1200 ± 560	80 ± 10	65.38 ± 5.68	21.23 ± 2.82
	[CuI(dmp)**3S**]	5.37 ± 0.65	4.04 ± 0.38	2330 ± 430	180 ± 80	86.49 ± 4.95	27.87 ± 4.24
	[CuNCS(dmp)**3S**]	2.12 ± 0.29	6.10 ± 0.73	5750 ± 320	140 ± 30	611.31 ± 17.67	45.28 ± 4.64
	cisplatin	2200 ± 820	3150 ± 450	4990 ± 670	3850 ± 430	39,040 ± 5450	43,310 ± 7210

## Data Availability

No new data were created or analyzed in this study. Data sharing is not applicable to this article.
